# Herpesviruses in etiopathogenesis of aggressive periodontitis: A meta-analysis based on case-control studies

**DOI:** 10.1371/journal.pone.0186373

**Published:** 2017-10-16

**Authors:** Fei Li, Ce Zhu, Feng-Ying Deng, May. Chun. Mei Wong, Hai-Xia Lu, Xi-Ping Feng

**Affiliations:** 1 Department of Preventive Dentistry, Ninth People’s Hospital, School of Medicine, Shanghai Jiao Tong University, Shanghai Key Laboratory of Stomatology, Shanghai, China; 2 Department of Endodontics, Shandong Province Hospital Affiliated to Shandong University, Jinan, Shandong, China; 3 Dental Public Health, Faculty of Dentistry, University of Hong Kong, Hong Kong, China; Ghent University, BELGIUM

## Abstract

**Objective:**

Previous studies have found that herpesviruses are associated with aggressive periodontitis (AgP). However, these findings are controversial. This meta-analysis was aimed at clarifying the association between herpesviruses and AgP.

**Methods:**

We identified eligible case–control studies evaluating the association between herpesviruses and AgP from PubMed and Embase databases in October 2015. Original data were extracted and quality assessment was done. Overall odds ratios (ORs) and 95% confidence intervals (CIs) were estimated. Random-effects model was determined. The stability was evaluated by sensitivity analysis. Finally, Egger’s funnel plot was used to investigate the publication bias.

**Results:**

Twelve case-control studies involving 322 patients and 342 controls were included in the present meta-analysis. The included case-control studies were assessed as high quality. The quantitative synthesis results for Epstein–Barr virus (EBV) showed significance (10 studies: *p* = 0.0008, OR = 6.11, 95% CI = 2.13–17.51); nevertheless, evidence of publication bias for EBV was considerable (EBV: Egger’s test, *p*<0.001). Human cytomegalovirus (HCMV) and Herpes simplex virus type 1 (HSV-1) had significant association with AgP (12 studies for HCMV: *p* = 0.009, OR = 3.63, 95% CI = 2.15–6.13; 4 studies for HSV-1: *p*<0.001, OR = 19.19, 95% CI = 4.16–79.06). Sensitivity analyses showed the results yielded consistency, and no significant publication bias was observed for HCMV. The association between Herpes simplex virus type 2 (HSV-2) and AgP was inconclusive (2 studies: *p* = 0.20, OR = 3.46, 95% CI = 0.51–23.51).

**Conclusion:**

This meta-analysis suggests that HCMV and HSV-1 are significantly associated with AgP. However, due to the heterogeneity among studies these conclusions should be cautiously interpreted. There is insufficient evidence to draw any conclusion between EBV, HSV-2 and AgP based on the currently limited data.

## Introduction

Aggressive periodontitis (AgP), characterized by early age of onset and tendency for familial aggregation, generally affects young patients, who show early and rapid periodontal breakdown with disproportionate amount of dental plaque in the lesion sites [1]. The etiopathogenesis of AgP involves complex interaction between multifarious microorganisms and the host immune system [2]. Bacteria have long been proposed as the causative and most important agents in the course of periodontal disease. However, the periodontal tissue destruction in AgP is usually site-specific, bilaterally symmetrical, occasionally breakout, and self-limited. These typical clinical manifestations of AgP cannot be well explained by bacterial infection alone [3]. Hence, scholars thought that pure bacterial aetiology of AgP may have been over-emphasised [4].

Herpesviruses have been implicated in the etiopathogenesis of human periodontal disease since 1990s. A meta-analysis trying to demonstrate the association between herpesviruses and chronic periodontitis have been published [5]. The etiopathogenesis of AgP differs from chronic periodontitis and the association between herpesviruses and AgP is still unclear. Numerous studies have investigated the association between herpesviruses and AgP [6–16]. However, the results of these studies remained controversial. Some researchers believed that herpesviruses do play a role in the etiopathogenesis of AgP, whereas others do not. Nibali *et al*. [9] failed to detect herpesviruses in any of the subgingival plaque samples from patients with AgP. Saygun *et al*. [17] reported no significant difference in copy numbers of herpesviruses between patients with AgP and periodontally healthy individuals.

Hence, the present study was conducted aiming at clarifying the association between occurrence of herpesviruses and risk of AgP by performing a meta-analysis based on available case–control studies (patients with AgP versus periodontally healthy individuals).

## Materials and methods

This study was in compliance with the Preferred Reporting Items for Systematic Reviews and Meta-Analyses (PRISMA) statement guidelines (More details were shown in S1 File). A review protocol did not exist.

### Search strategy

Fei Li and Ce Zhu conducted computerized search of PubMed and Embase databases in October 2015 to retrieve relevant case–control studies investigating the association between herpesviruses and AgP. Terms employed for literature retrieve included: (1) “virus”, “herpesvir*”, “EBV”, “CMV”, “HCMV”, “HHV”, “HSV”, “Epstein–Barr virus”, “cytomegalovirus”, “herpes simplex virus”, “Human herpesvirus”; and (2) “aggressive periodontitis”, “parodontopathy”, “periodontal disease”, “periodontal”, “paradontosis”. The literature search was performed on English-language articles without any other restrictions (Electronic search strategy of PubMed database was shown on S2 File). Furthermore, we conducted a manual search on the reference lists of the eligible articles.

### Inclusion and exclusion criteria

Studies were considered eligible if they met the following inclusion criteria: (1) studies were designed as case–control studies (patients with AgP vs periodontally healthy controls); (2) studies investigated the association between herpesviruses and AgP; (3) study samples were collected from these locations: biopsy, subgingival plaque, or gingival crevicular fluid; (4) the methods for herpesviruses’ detection were as follows: polymerase chain reaction (PCR), nested PCR, multiplex PCR, or real-time PCR; (5) all participants in the case and control groups were systemically healthy; and (6) the original articles provided available data, or we can obtain available data from the authors by inquiring email.

Studies were excluded if they met the following exclusion criteria: (1) studies were not of case–control design; (2) participants with systemic disease were involved in the case and control groups; (3) study samples were collected from saliva; (4) no available data could be obtained; and (5) study population were duplicate.

### Study selection

Potential inclusion was considered by screening title and abstract of all retrieved articles. Then full text was obtained for possible qualified articles. The same two reviewers independently assessed full text according to inclusion and exclusion criteria, eligible studies were submitted for final inclusion.

### Data extraction

Data extraction was conducted in duplicate from included studies by the same two reviewers. The extracted data included first author, publication year, race of the study population, gender, mean age, number of patients and controls, sampling method, sample type, method of sample analyse, and detection rate of each herpesvirus. Controversial issues were resolved through discussion. Authors were directly contacted if crucial data was missing in original articles.

### Quality assessment

The methodological quality of each studies were assessed independently by the same two reviewers using the Newcastle–Ottawa Scale (NOS) [18]. Total quality scores of the NOS ranges from 0 points to 9 points. Meanwhile, a higher score manifested better methodological quality. Studies with 7 points or higher were considered to be of high quality.

### Statistical analysis

All statistical analysis was carried out using RevMan version 5.1 (The Nordic Cochrane Centre, The Cochrane Collaboration, Copenhagen, Denmark) and STATA version 12.0 (StataCorp, College Station, TX, USA) software. We estimated ORs and corresponding 95% CIs to measure the association between herpesviruses and risk of AgP. We didn’t have the assumption that all studies share a common effect, thus random-effects model was determined for all the quantitative synthesises. Additionally, the stability of the quantitative synthesis was evaluated by sensitivity analysis. One study was omitted sequentially, then the calculated combined OR for the remaining studies were assessed. We explored potential publication bias by constructing funnel plots and plot asymmetry test. Egger’s funnel plot asymmetry test (linear regression method) was performed to investigate the publication bias. *P* value less than 0.05 was considered as statistically significant and all test were two-sided.

## Results

### Description of the studies

**Fig 1:**
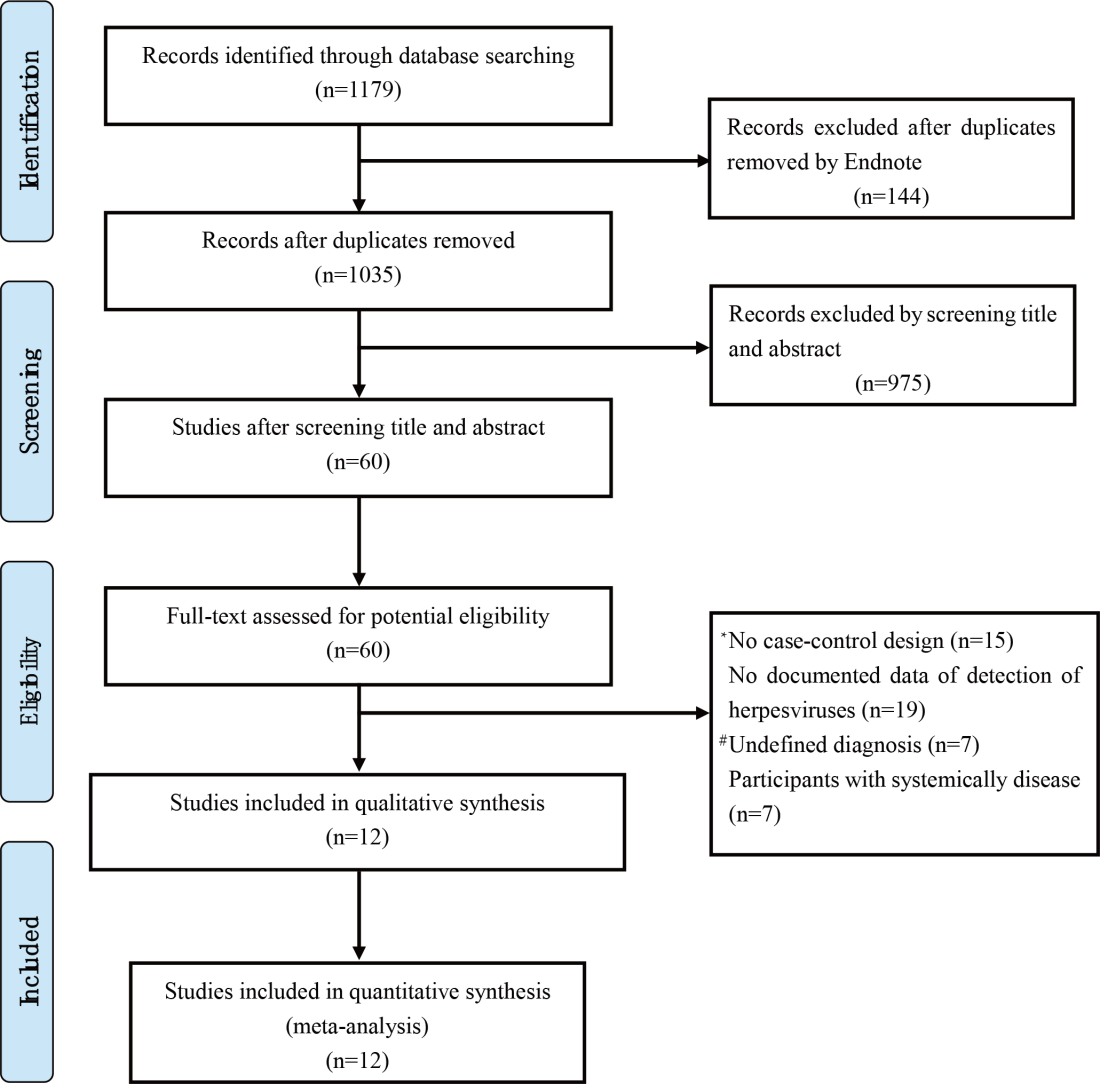
Flow chart for the study selection process. # Undefined diagnosis: The diagnosis of patients is not appropriate or not clear. * No case-control design: Study is not design as case-control style.

The process of study selection was shown in Fig 1. 1179 records were initially identified from PubMed and Embase databases. 1035 records were left after duplicates removed. Moreover, we excluded 975 non-relevant records by screening title and abstract. Then full texts of the remaining 60 articles were assessed in detail. Twelve eligible studies were included for meta-analysis according to the inclusion and exclusion criteria. Articles excluded from this step and the exclusion reasons were presented in S1 Table. Characteristics of the included studies were summarised in Table 1. There are 322 AgP patients and 342 periodontally healthy controls in our meta-analysis. The quality assessment scores using the NOS were also shown in Table 1, all the included studies were considered to be of high quality (The details of the assessment of the quality of the studies by NOS was shown on S3 File).

**Table 1:** The characteristics of studies included in the meta-analysis.

Studies	Race	Participants(cases/controls)		Materials and Methods			Herpesviruses prevalence		Quality assessment
		Sample size	Mean age	Sample type	Sampling method	Analysis method	Cases	Controls	
Michalowicz et al, 2000 [8]	Jamaica	15/65	N/A	SP	Paper point	nested PCR	EBV(+)5/15,HCMV(+)11/15	EBV(+)11/65,HCMV(+)14/65	7
Yapar et al, 2003 [10]	Turkey	17/16	24.05/24.12	SP	Curette	PCR	EBV(+)12/17,HCMV(+)11/17	EBV(+)1/16,HCMV(+)0/16	8
Saygun et al, 2004 [19]	Turkey	18/16	24.1/24.1	SP	Curette	Q-PCR	EBV(+)13/18,HCMV(+)13/18, HSV-1(+) 14/18, HSV-2(+) 13/18	EBV(+)1/16,HCMV(+)0/16,HSV-1(+) 0/16, HSV-2(+) 0/16	8
Kubar et al, 2005 [20]	Turkey	16/15	24.1/24.1	SP	Curette	Q-PCR	HCMV(+)11/16	HCMV(+)0/15	8
Betero et al, 2007 [11]	Colombia	10/22	24.3/31.2	SP	Paper point	nested PCR	HCMV(+)4/10	HCMV(+)4/22	7
Retola et al, 2008 [12]	Italy	11/13	40.9/25.8	BS	Biopsies	nested PCR	EBV(+)6/11,HCMV(+)0/11,HHV-7(+)7/11	EBV(+)15/16,HCMV(+)16/16,HHV-7(+)8/13	7
Imbronito et al, 2008 [13]	Brazil	30/30	27.3/28.1	SP	Paper point	nested PCR	EBV(+)10/30,HCMV(+)18/30	EBV(+)0/30,HCMV(+)17/30	8
Nibali et al, 2009 [9]	UK	80/40	32.3/50.3	SP	Curette	realtime-PCR	EBV(+)6/80,HCMV(+)0/80	EBV(+)4/40,HCMV(+)0/40	7
Das et al, 2012 [14]	India	25/25	N/A	SP	Curette	Q-PCR	EBV(+)8/25,HCMV(+)3/25, HSV-1(+) 20/25, HSV-2(+) 2/25	EBV(+)2/25,HCMV(+)2/25,HSV-1(+) 3/25, HSV-2(+) 1/25	7
Sharma et al, 2012 [15]	India	20/20	29.6/36.5	SP	Curette	PCR	EBV(+)9/20,HCMV(+)9/20	EBV(+)0/20,HCMV(+)2/20	8
Stein et al, 2013 [16]	Germany	65/65	35.4/40.0	SP	Paper point	realtime-PCR	EBV(+)7/65,HCMV(+)1/65, HSV-1(+) 1/65	EBV(+)9/65,HCMV(+)1/65,HSV-1(+) 1/65	8
Sharma et al, 2015[21]	India	15/15	24.6/23.6	SP	Curette	PCR	EBV(+)6/15,HCMV(+)7/15	EBV(+)1/15,HCMV(+)1/15	8

Abbreviations: N/A, not applicable; SP, subgingival plaque; BS, biopsy specimen; PCR, polymerase chain reaction.

### Quantitative synthesis

**Fig 2:**
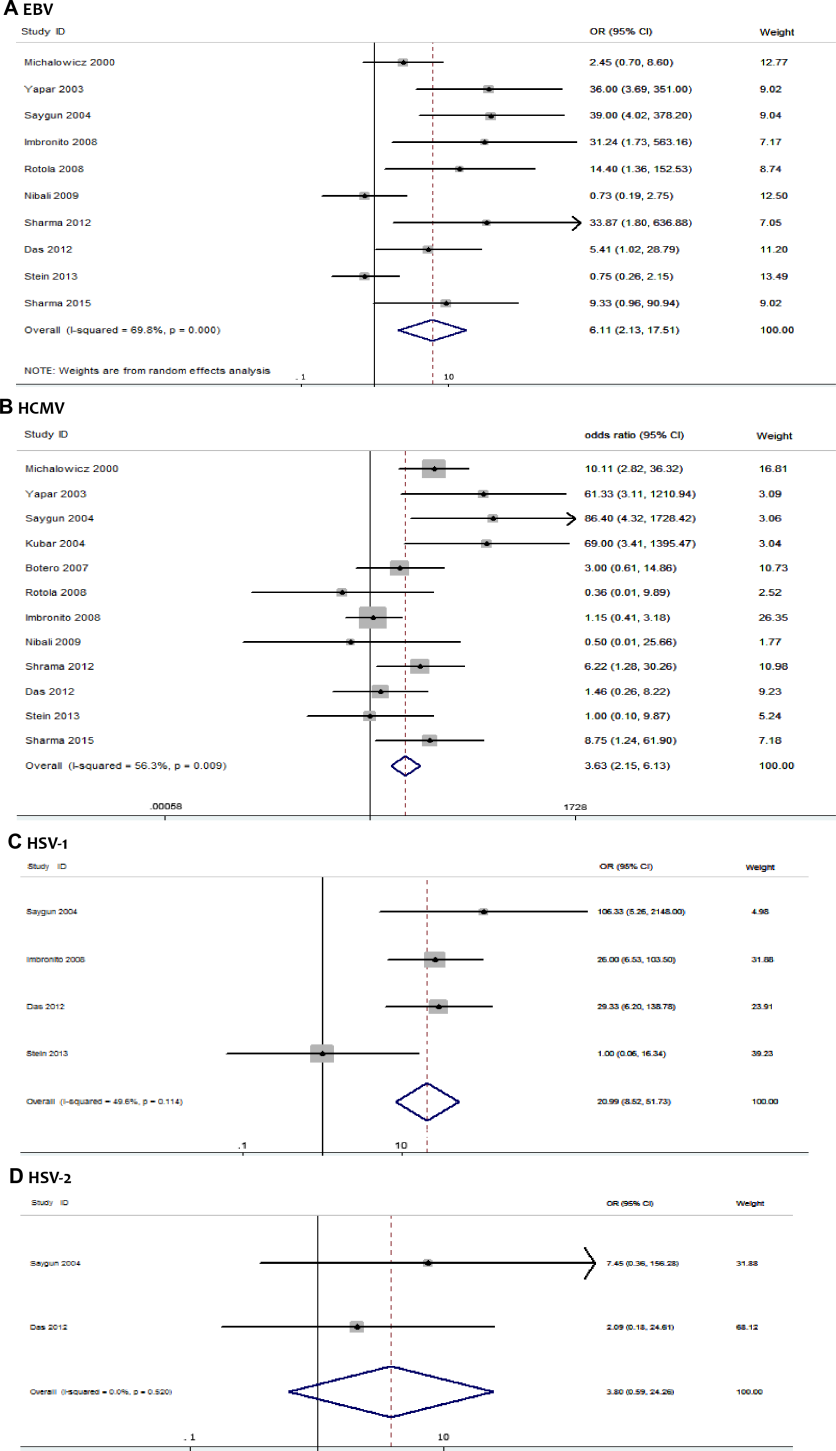
Forest plots of the association between herpesviruses and risk of AgP. (A) EBV and AgP risk, (B) HCMV and AgP risk, (C) HSV-1 and AgP risk, (D) HSV-2 and AgP risk.

Forest plots of the association between occurrence of herpesviruses and risk of AgP were shown in Fig 2. Quantitative synthesis was conducted separately by herpesvirus type. Association between EBV and AgP was analysed in ten studies, comprising 296 AgP patients and 305 periodontally healthy controls. The overall result based on random-effects model showed a significance between EBV and risk of AgP (10 studies: *p* = 0.0008, OR = 6.11, 95% confidence interval CI = 2.13–17.51) (Fig 2A).

12 studies involving 322 AgP patients and 342 periodontally healthy controls were examined to investigate the association between HCMV and risk of AgP. The overall result based on random-effects model showed that HCMV was associated with significantly increased risk of AgP (*p* = 0.009, OR = 3.63, 95% CI = 2.15–6.13) (Fig 2B).

Four eligible studies were examined to investigate the association between HSV-1 and AgP risk. The overall result based on random -effects model showed that HSV-1 was associated with significantly increased risk of AgP (*p*<0.001, OR = 19.19, 95% CI = 4.16–79.06) (Fig 2C).

Two eligible studies were examined to investigate the association between HSV-2 and risk of AgP. The pooled OR was estimated based on random-effects model (*p* = 0.20, OR = 3.46, 95% CI = 0.51–23.51) (Fig 2D). The association between HSV-2 and AgP was inconclusive due to insufficient data.

Only one study included covered the association between HHV-7 and risk of AgP (OR = 1.09, 95% CI = 0.21–5.76). VZV, HHV-6, and HHV-8 haven’t been reported in included studies.

### Sensitivity analysis

**Fig 3:**
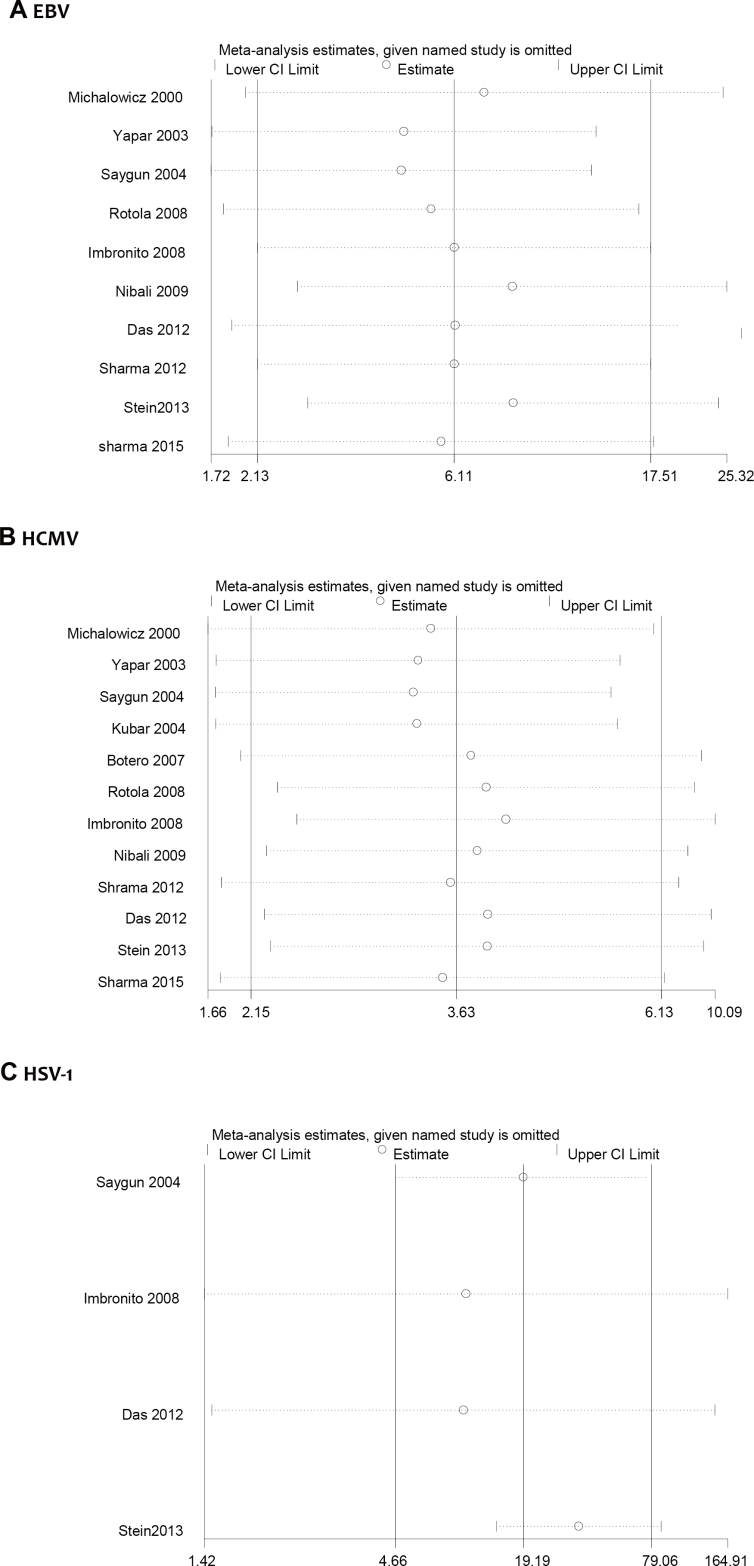
Sensitivity analysis of the association between herpesviruses and risk of AgP. (A) EBV and AgP risk, (B) HCMV and AgP risk, (C) HSV-1 and AgP risk.

Fig 3 showed the sensitivity analysis results of the associations between EBV, HCMV, HSV-1 and AgP. We conducted sensitivity analysis by omitting one study each time. The overall results yielded consistent results. In other words, no single study changed the overall ORs significantly, proving that the results were reliable. For the number limitation of articles included, sensitivity analysis for rest members of the herpesvirus family were not assessed.

### Publication bias

**Fig 4:**
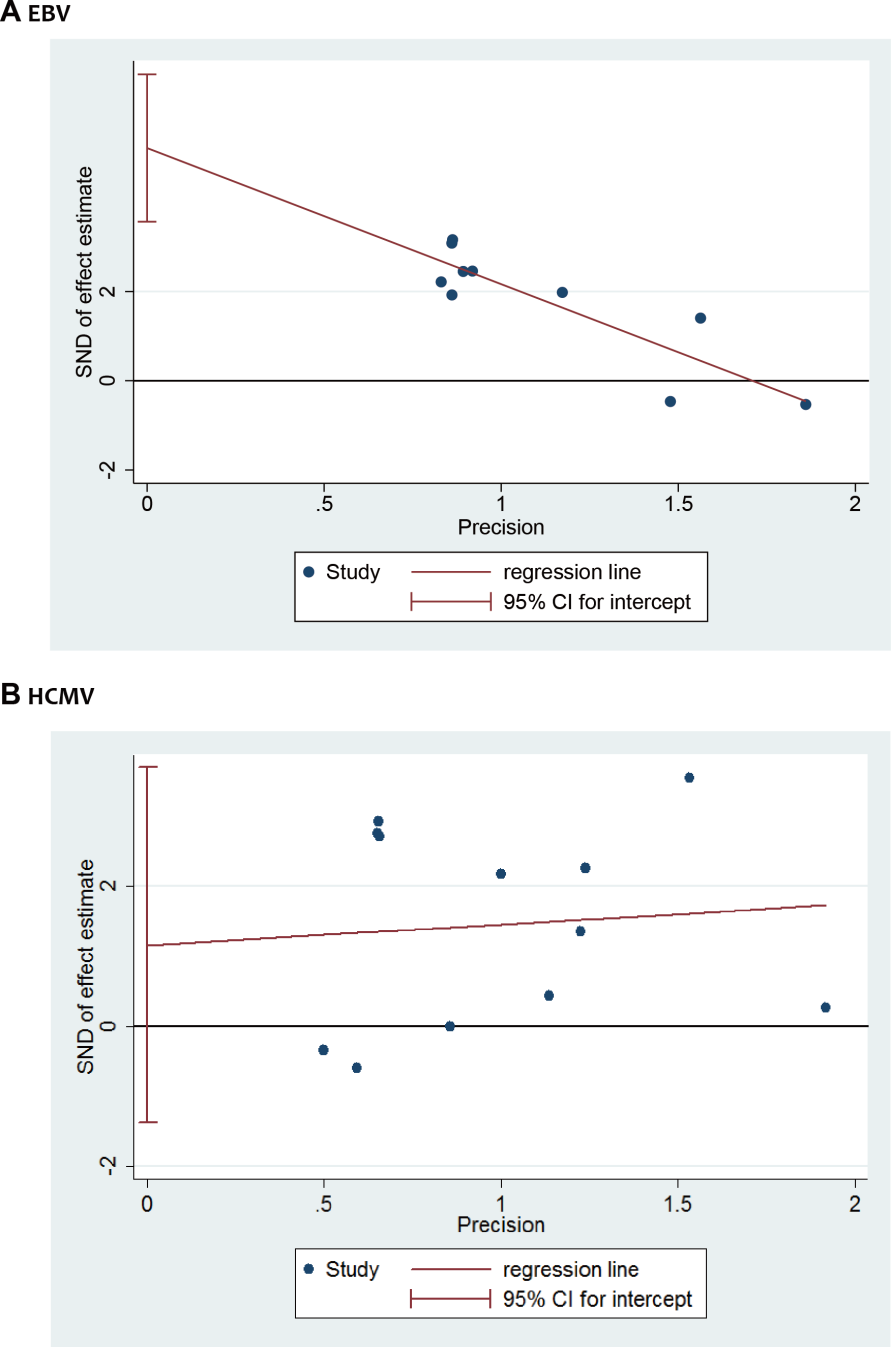
Egger’s funnel plot of the association between herpesviruses and risk of AgP. (A) EBV and AgP risk, (B) HCMV and AgP risk, (C) HSV-1 and AgP risk.

Publication bias was treatedusing Egger’s funnel plot asymmetry test (linear regression method) according to herpesvirus type. Significant publication bias for EBV was detected using Egger’s funnel plot asymmetry test (EBV: Egger’s test, *p* < 0.001) (Fig 4A). No significant publication bias was detected for HCMV (HCMV: Egger’s test, *p* = 0.332). For the number limitation of articles included, sensitivity analysis for rest members of the herpesvirus family were not assessed.

## Discussion

Aggressive periodontitis is a high-risk and multi-factor disease which would result in the early loss of human teeth. The conventional pure bacterial pathogenicity theory of periodontal diseases was somewhat too weak to cover all the manifestation of AgP. Since mid 1990s when herpesviruses emerging as putative pathogens in periodontal diseases [22], more and more evidence convince that the presence of herpesviruses in the periodontal environment is associated with the occurrence and severity of AgP [10, 12–15, 19]. And compared with other types of periodontal diseases, AgP seems to have a closer connection with herpesviruses. The typical clinical manifestation of AgP like little plaque formation at sites with rapid periodontium destruction could be better explained by alteration between active and latent periods of herpesvirus infection [23]. However, most of the data supporting these views come from the same research team, and contradictory reports have also been published. Some groups have reported weak connection even opposite results between AgP and herpesviruses [9, 11, 19]. Therefore, it is necessary to give overall estimations on the association between herpesviruses and AgP based on existing research data.

Several recent reviews [4, 23–26] have summarized the published findings on interaction between herpesviruses and AgP. Nevertheless, no quantitative synthesis analysis have been reported. Hence, this meta-analysis may help to provide more convincible evidence. Twelve studies were included in current meta-analysis. In five of twelve studies, HCMV genomes were detected in >50% of infected sites [8, 10, 13, 19, 27], while two studies reported negative results in all the samples [9, 12]. Control groups generally showed a low frequency of virus detection [8, 11, 14–16, 21]. Nevertheless, two control group studies reported HCMV occurrence higher than 50% [12, 13], whereas four did not detect HCMV in any of the infected sites [9, 10, 19, 27]. As for the detection of EBV, three out of ten studies reported a detection frequency of >50% in the infected site of AgP patients [10, 12, 19]. The other seven studies only detected EBV genomes in <45% of infected sites [8, 9, 13–16, 21]. Studies on periodontally healthy volunteers generally reported a low detection frequency of EBV, except for one study that detected EBV genomes in >50% of infected sites [12]. Only three included studies reported the occurrence of HSV, two of which detected HSV genomes in >50% of infected sites of AgP patients [14, 19], and in fewer sites in the periodontally healthy group.

All the included studies reported adequate definition for patients in case group. The diagnosis criteria for “aggressive periodontitis” was based on the 1999 Consensus Classification [28] except for the study by Michalowicz et al [8]. This study was submitted to final inclusion because the diagnosis criteria for case group in this study was in accordance with the 1999 Consensus Classification.

From the current meta-analysis, we concluded that HCMV and HSV-1 have significant association with increased risk of AgP. Both sensitivity analysis and publication bias test showed that the overall estimates were comparatively robust. Nevertheless, the significant pooled OR from quantitative synthesis for EBV was inconclusive because of considerable publication bias was found. Insufficient evidence was obtained to support the association between HSV-2 and HHV-7 and AgP since only two eligible studies for HSV-2 and one for HHV-7 were included. Additional high-quality, large scale studies are needed to achieve an unprejudiced conclusion. The rest members of the herpesvirus family were not covered in the studies included in our meta-analysis.

The exact pathogenic mechanisms of herpesviruses in periodontitis remain unclear. To the best of our knowledge, herpesviruses intervene in the inflammatory process by altering the immune mechanism and immune response in direct or indirect ways. Herpesviruses may exert their pathogenicity by a direct effect on different cells in the periodontium, virus-induced alteration of host immune defences, and enhancement of virulence of pathogenic bacteria [25]. Wara-Aswapati described a hypothesis that, HCMV plays a role in the pathogenesis of periodontal disease by the ability of its immediate early proteins to strongly transactivate IL-1β gene expression[29]. The most popular view in recent years is the pathogenic synergy between periodontopathic bacteria and herpesviruses [23, 30]. Herpesvirus infection predisposes the periodontal tissues to secondary infections by altering the adherence, chemotaxis, phagocytosis and bactericidal activity of polymorphonuclear leucocytes [26]. Also, periodontopathic bacteria might activate periodontal herpesviruses through inflammation-inducing factors. Recent studies found that *Porphyromonas gingivalis* has the potential to trigger EBV by increasing the activity of the BZLF1 gene, which encodes the key protein for the transition from latency to the lytic replication cycle [31, 32]. Another study found that the co-infection of *Porphyromonas gingivalis* and EBV increase the gingival crevicular fluid visfatin levels, which might stimulate the expression of matrix-degrading enzymes and the breakdown of periodontal tissues [33].

Several limitations should be acknowledged in our meta-analysis. First, significant heterogeneity was detected among included studies in quantitative synthesis for HCMV and HSV-1. This may be caused by the differences on participants (age, gender, ethnic groups), sampling methods (Paper point, Curette, Biopsies), sample type (Subgingival plaque) and analysis methods (PCR, nested PCR, realtime-PCR, Q-PCR). However, we were unable to conduct subgroup analysis owing limited data. The conclusions should be cautiously interpreted. Second, confounding effect of age exist as a challenging task, although we have extracted the participants’ age distributions from each included study. Third, all included studies were published in English, leading to the limitation of data source and possibility of publication bias. According to the results of Egger’s funnel plot and Begg’s rank correlation test, publication bias for EBV was considerable, which means the result for EBV and risk of AgP might be inconclusive. However, there was no observable publication bias for HCMV and HSV-1. Last, the conclusion of this study was based on 12 independent studies and 664 subjects, which might be insufficient to quantify the risk estimate reliably. Especially, only four studies were included for HSV-1, which might not sufficient enough for a robust result.

In conclusion, the results from the current meta-analysis suggest that HCMV and HSV-1 are associated with significantly increased risk of AgP. Sensitivity analysis showed similar results. There was no indication of publication bias for HCMV and HSV-1, substantiating the robustness of our findings. Nevertheless, significant publication bias was observed for EBV, and the significant overall OR for EBV was inconclusive. These findings should be interpreted cautiously in view of the considerable heterogeneity among the included studies concerning HCMV and HSV-1. This study might provide a better understanding of the association between herpesviruses and AgP. Future research should be conducted to investigate co-infection with herpesviruses and bacteria and associated host responses in the development of periodontitis.

## Supporting information

S1 Table Articles excluded after critical appraisal. * with systematically diseases: Patients with systemic diseases or infections. # Undefined diagnosis: The diagnosis of patients is not appropriate or not clear. ^ No case-control design: Study is not design as case-control style.
(PDF)

S1 File PRISMA checklist. (DOC)

S2 File Electronic search strategy of PubMed database. (DOCX)

S3 File Quality score assessment. (DOCX)
