# Supplementary material for: Herpesviruses in etiopathogenesis of aggressive periodontitis: A meta-analysis based on case-control studies
Source: PLoS One. 2017 Oct 16;12(10):e0186373. doi: 10.1371/journal.pone.0186373 (PMC5643052; doi:10.1371/journal.pone.0186373)
Supplement: S1 Table — * with systematically diseases: Patients with systemic diseases or infections. # Undefined diagnosis: The diagnosis of patients is not appropriate or not clear. ^ No case-control design: Study is not design as case-control style. (PDF) [file pone.0186373.s001.pdf]

S1 Table . Articles Excluded after Critical Appraisal

Studies	Title	Reasons for exclusion
Winters et al, 1984	Humoral immune responses to adenoviruses, herpes virus type 1, and Candida albicans in sera of dental patients with oral neoplastic and periodontal diseases	With systemically diseases*
Madinier et al, 1992	Epstein-Barr virus DNA detection in gingival tissues of patients undergoing surgical extractions	With systemically diseases
Contreras et al, 1996	Mammalian viruses in human periodontitis	No case-control design^
Parra et al, 1996	Detection of human viruses in periodontal pockets using polymerase chain reaction	No case-control design
Contreras et al, 1998	Active cytomegalovirus infection in human periodontitis	No documented data of detection of herpesviruses
Contreras et al, 1999	Herpesvirus infection of inflammatory cells in human periodontitis	No documented data of detection of herpesviruses
Contreras et al, 1999	Relationship between herpesviruses and adult periodontitis and periodontopathic bacteria	No case-control design
Contreras et al, 2000	Herpesviruses in periodontal pocket and gingival tissue specimens	Undefined diagnosis#
Mardirossian et al, 2000	Herpesviruses 6, 7 and 8 in HIV- and non-HIV-associated periodontitis	With systemically diseases
Ting et al, 2000	Herpesvirus in localized juvenile periodontitis	No case-control design
Contreras et al, 2001	Herpesviruses in HIV-periodontitis Dentistry	With systemically diseases
Contreras et al, 2001	Typing of herpes simplex virus from human periodontium	No case-control design
Kamma et al, 2001	Herpes viruses and periodontopathic bacteria in early-onset periodontitis	No case-control design
Slots et al, 2002	Detection of human viruses in patients with chronic periodontitis and the relationship between viruses and clinical parameters	No documented data of detection of herpesviruses
Cassai et al, 2003	HHV-6, HHV-7, HHV-8 in gingival biopsies from chronic adult periodontitis patients. A case-control study	No documented data of detection of herpesviruses
Slots et al, 2003	The herpesvirus-Porphyromonas gingivalis-periodontitis axis	No documented data of detection of herpesviruses
Idesawa et al, 2004	Detection of Epstein-Barr virus in saliva by real-time PCR	No case-control design
Konstantinidis et al, 2004	Relationship between Porphyromonas gingivalis, Epstein-Barr virus infection and reactivation in periodontitis	No case-control design
Surang et al, 2004	Association between human herpesviruses and the severity of periodontitis	No case-control design
Surang et al, 2004	Prevalence of cytomegalovirus, human herpesvirus-6, and Epstein-Barr virus in periodontitis patients and healthy subjects in the Thai population	Undefined diagnosis

Klemenc et al, 2005	Prevalence of some herpesviruses in gingival crevicular fluid	Undefined diagnosis
Konstantinidis et al, 2005	Real-time polymerase chain reaction quantification of Epstein--Barr virus in chronic periodontitis patients	No documented data of detection of herpesviruses
Kubar et al, 2005	Real-time polymerase chain reaction quantification of human cytomegalovirus and Epstein-Barr virus in periodontal pockets and the adjacent gingiva of periodontitis lesions	No case-control design
Saygun et al, 2005	Periodontitis lesions are a source of salivary cytomegalovirus and Epstein-Barr virus	No case-control design
Radvar et al, 2006	Herpesviruses at human aggressive periodontitis sites	No case-control design
WU et al, 2006	Infection frequency of Epstein-Barr virus in subgingival samples from patients with different periodontal status and its correlation with clinical parameters	No documented data of detection of herpesviruses
Yildirim et al, 2006	Detection and quantification of herpesviruses in Kostmann syndrome periodontitis using real-time polymerase chain reaction_ a case report	With systemically diseases
Watanabe et al, 2006	EBV-1 and HCMV in aggressive periodontitis in Brazilian patients	No case-control design
WU et al, 2007	Correlation between infections with different genotypes of human cytomegalovirus and Epstein-Barr virus in subgingival samples and periodontal status of patients	No documented data of detection of herpesviruses
Botero et al, 2008	Comparison of nested polymerase chain reaction (PCR), real-time PCR and viral culture for the detection of cytomegalovirus in subgingival samples	Undefined diagnosis
Chalabi et al, 2008	EBV and CMV in chronic periodontitis a prevalence study	No documented data of detection of herpesviruses
Grande et al, 2008	Herpes viruses in periodontal compromised sites comparison between HIV-positive and -negative patients	With systemically diseases
Imbronito et al, 2008	Detection of Epstein-Barr virus and human cytomegalovirus in blood and oral samples comparison of three sampling methods	No case-control design
Pia et al, 2008	Human cytomegalovirus and Epstein-Barr virus in apical and marginal periodontitis a role in pathology	Undefined diagnosis
Saygun et al, 2008	Quantitative analysis of association between herpesviruses and bacterial pathogens in periodontitis	No documented data of detection of herpesviruses
Sheila et al, 2008	Occurrence of herpes simplex virus 1 and three periodontal bacteria in patients with chronic periodontitis and necrotic pulp	No documented data of detection of herpesviruses
Chalabi et al, 2009	Herpesviruses in chronic and aggressive periodontitis patients in an Indian population	No case-control design
Dawson et al, 2009	Salivary levels of Epstein-Barr virus DNA correlate with subgingival levels, not severity of periodontitis	No documented data of detection of herpesviruses

Dophus et al, 2009	Real-time polymerase chain reaction to determine the prevalence and copy number of epstein-barr virus and cytomegalovirus DNA in subgingival plaque	No documented data of detection of herpesviruses
Grenier et al, 2009	Detection of herpetic viruses in gingival crevicular fluid of patients suffering from periodontal diseases prevalence and effect of treatment	Undefined diagnosis
Chalabi et al, 2010	Periodontopathic bacteria and herpesviruses in chronic periodontitis	No documented data of detection of herpesviruses
Sabrina et al, 2011	Relationship between herpesviruses and periodontopathogens in patients with HIV and periodontitis	With systemically diseases
Saygun et al,2011	Salivary infectious agents and periodontal disease status	No documented data of detection of herpesviruses
Thomasini et al, 2011	Correlation of cytomegalovirus and human herpesvirus 7 with CD3+ and CD3+CD4+ cells in chronic periodontitis patients	No documented data of detection of herpesviruses
Ayako et al,2013	Higher Prevalence of Epstein -Barr Virus DNA in Deeper Periodontal Pockets of Chronic Periodontitis in Japanese Patients	No documented data of detection of herpesviruses
Leon et al,2013	The prevalence of human herpes viruses in the saliva of chronic periodontitis patients compared to oral health providers and healthy controls	No documented data of detection of herpesviruses
Kato et al,2014	Prevalence and quantitative analysis of Epstein -Barr virus DNA and Porphyromonas gingival is associated with Japanese chronic periodontitis patients	No documented data of detection of herpesviruses
Petrovic et al,2014	Detection of herpes simplex virus type 1 in gingival crevicular fluid of gingival sulcus periodontal pocket using polymerase chain reaction	Undefined diagnosis
