# Supplementary material for: Herpesviruses in etiopathogenesis of aggressive periodontitis: A meta-analysis based on case-control studies
Source: PLoS One. 2017 Oct 16;12(10):e0186373. doi: 10.1371/journal.pone.0186373 (PMC5643052; doi:10.1371/journal.pone.0186373)
Supplement: S2 File — (DOCX) [file pone.0186373.s003.docx]

(((((((((((((viruses[Title/Abstract]) OR virus[Title/Abstract]) OR herpesvir*[Title/Abstract]) OR EBV[Title/Abstract]) OR CMV[Title/Abstract]) OR HCMV[Title/Abstract]) OR HHV[Title/Abstract]) OR HSV[Title/Abstract]) OR Epstein-Barr virus[Title/Abstract]) OR cytomegalovirus[Title/Abstract]) OR herpes simplex virus[Title/Abstract]) OR Human herpesvirus[Title/Abstract])) AND ((((((periodontal disease[Title/Abstract]) OR paradontosis[Title/Abstract]) OR parodontopathy[Title/Abstract]) OR periodontal[Title/Abstract]) OR periodontium[Title/Abstract]) OR periodontitis[Title/Abstract])
