# Supplementary material for: Herpesviruses in etiopathogenesis of aggressive periodontitis: A meta-analysis based on case-control studies
Source: PLoS One. 2017 Oct 16;12(10):e0186373. doi: 10.1371/journal.pone.0186373 (PMC5643052; doi:10.1371/journal.pone.0186373)
Supplement: S3 File — (DOCX) [file pone.0186373.s004.docx]

**Quality assessment**

The same two reviewers independently assessed the quality of each studies using the Newcastle–Ottawa Scale (NOS). Total quality scores of the NOS ranges from 0 points to 9 points. Meanwhile, a higher score manifested better methodological quality. Studies with 7 points or higher were considered to be of high quality.

Wells GA, Shea B, O’Connell D, Peterson J, Welch V, M Losos, et al. (2000) The Newcastle-Ottawa Scale (NOS) for assessing the quality of nonrandomized studies in meta-analyses. Available at http://www.ohri.ca/programs/clinical_epidemiology/nosgen.pdf.

| Studies | Selection | Comparability | Exposure | Total |
| --- | --- | --- | --- | --- |
| Michalowicz 2000 | 4 | 1 | 2 | 7 |
| Yapar 2003 | 4 | 2 | 2 | 8 |
| Saygun 2004 | 4 | 2 | 2 | 8 |
| Kubar2005 | 4 | 2 | 2 | 8 |
| Betero 2007 | 4 | 1 | 2 | 7 |
| Retola 2008 | 4 | 1 | 2 | 7 |
| Imbronito 2008 | 4 | 2 | 2 | 8 |
| Nibali 2009 | 4 | 1 | 2 | 7 |
| Das 2012 | 4 | 1 | 2 | 7 |
| Sharma 2012 | 4 | 2 | 2 | 8 |
| Stein 2013 | 4 | 2 | 2 | 8 |
| Sharma 2015 | 4 | 2 | 2 | 8 |
